# Supplementary material for: Reconciling Mining with the Conservation of Cave Biodiversity: A Quantitative Baseline to Help Establish Conservation Priorities
Source: PLoS One. 2016 Dec 20;11(12):e0168348. doi: 10.1371/journal.pone.0168348 (PMC5173368; doi:10.1371/journal.pone.0168348)
Supplement: S1 Dataset — (ZIP) [file pone.0168348.s002.zip › Taxa/Serra Norte/SN_2013/N4WS/Lista N4WS-38.pdf]

CAVIDADE N4WS-0038

Classe	Ordem	Fam/Outros	Gên/Outros	Espécie	S-U	U-S	Zon.
Arachnida	Araneae	Anyphaenidae		jovem	X		E
Arachnida	Araneae	Ctenidae		jovem	3	4	E
Arachnida	Araneae	Drymusidae		jovem	X		E
Arachnida	Araneae	Filistatidae		jovem	X		E
Arachnida	Araneae	Mysmenidae		jovem	X		E
Arachnida	Araneae	Pholcidae	*Leptopholcus*	sp.1	X		E
Arachnida	Araneae	Pholcidae	*Mesabolivar*	*eberhard*	X		E
Arachnida	Araneae	Pholcidae		jovem		X	E
Arachnida	Araneae	Salticidae	*Amphidraus*	sp.1		X	E
Arachnida	Araneae	Salticidae	*Freya*	sp.1		X	E
Arachnida	Araneae	Scytodidae	*Scytodes*	jovem		1	E
Arachnida	Araneae	Tetragnathidae		jovem	X		E
Arachnida	Araneae	Theraphosidae		sp.1	1		E
Arachnida	Araneae	Theridiidae		jovem		X	E
Arachnida	Opiliones	Cosmetidae		sp.1		1	E
Arachnida	Opiliones	Sclerosomatidae		jovem		X	E
Arachnida	Opiliones	Stygnidae	*Protimesius*	*laevis*	1		E
Arachnida	Pseudoscorpiones	Olpiidae		sp.1	X		E
Arachnida	Schizomida	Hubbardiidae	aff. *Rowlandius*	sp.1		X	E
Diplopoda	Polyxenida	Hypogexenidae		sp.1	X		E
Entognatha	Collembola	Entomobryidae		sp.6		X	E
Entognatha	Collembola	Isotomidae	*Isotomiella*	*nummulifer*	X		E/P/A
Entognatha	Collembola	Paronellidae		sp.1	X		E
Entognatha	Collembola	Paronellidae		sp.2	X		E
Entognatha	Diplura	Campodeidae		sp.1		X	E
Insecta	Blattodea	Polyphagidae		jovem	2	2	E
Insecta	Coleoptera			jovem		X	E
Insecta	Diptera	Ceratopogonidae		sp.	X		E
Insecta	Diptera	Chironomidae		sp.		X	E
Insecta	Diptera	Culicidae	*Culex*	sp.		X	E
Insecta	Diptera	Dolichopodidae		sp.	X		E
Insecta	Diptera	Ephydridae		sp.	X		E
Insecta	Diptera	Limoniidae		sp.	X		E
Insecta	Diptera	Sciaridae	*Bradysia*	sp.	X		E
Insecta	Diptera	Tipulidae	Tipulinae	sp.	X	X	E
Insecta	Heteroptera	Reduviidae	Reduviinae	jovem	16		E
Insecta	Homoptera	Cixiidae		jovem		X	E
Insecta	Hymenoptera	Formicidae	*Crematogaster*	sp.1	X		E
Insecta	Hymenoptera	Formicidae	*Crematogaster*	sp.3		X	E
Insecta	Hymenoptera	Formicidae	*Nylanderia*	sp.2	X		E
Insecta	Hymenoptera	Formicidae	*Pachycondyla*	*striata*	X		E
Insecta	Hymenoptera	Formicidae	*Pheidole*	sp.2	X		E
Insecta	Hymenoptera	Pemphredonidae		sp.1		X	E
Insecta	Isoptera	Termitidae	*Nasutitermes*	sp.		X	E
Insecta	Lepidoptera	Noctuoidea		sp.1		15	E
Insecta	Lepidoptera	Noctuoidea		sp.2	X	X	E
Insecta	Lepidoptera	Noctuoidea		sp.3		X	E
Insecta	Neuroptera	Myrmeleontidae		jovem	1	2	E
Insecta	Orthoptera	Phalangopsidae	*Paraclodes*	sp.	3		E

Insecta	Psocoptera	Lepidopsocidae	*Nepticulomima*	sp.1	X		E
Insecta	Psocoptera	Psocomorpha		jovem		X	E
Gastropoda	Pulmonata	Systrophiidae	*Happia*	sp.		X	E
Amphibia	Anura	Strabomantidae	*Pristimantis*	cf. *fenestratus*	3		E
Mammalia	Chiroptera	Emballonuridae	*Peropteryx*	sp.	3	2	E
Reptilia	Squamata	Gekkonidae	*Thecadactylus*	sp.	1		E
Reptilia	Squamata	Gymnophthalmidae	aff. *Neusticurus*	sp.		1	E
